# L-RAPiT: A Cloud-Based Computing Pipeline for the Analysis of Long-Read RNA Sequencing Data

**DOI:** 10.3390/ijms232415851

**Published:** 2022-12-13

**Authors:** Theodore M. Nelson, Sankar Ghosh, Thomas S. Postler

**Affiliations:** Department of Microbiology & Immunology, Vagelos College of Physicians & Surgeons, Columbia University Irving Medical Center, New York, NY 10032, USA

**Keywords:** *LINC00173*, alternative splicing, bioinformatics, computational genomics, next-generation sequencing, software, PacBio, Oxford Nanopore, long-read sequencing, RNA sequencing, RNA-seq

## Abstract

Long-read sequencing (LRS) has been adopted to meet a wide variety of research needs, ranging from the construction of novel transcriptome annotations to the rapid identification of emerging virus variants. Amongst other advantages, LRS preserves more information about RNA at the transcript level than conventional high-throughput sequencing, including far more accurate and quantitative records of splicing patterns. New studies with LRS datasets are being published at an exponential rate, generating a vast reservoir of information that can be leveraged to address a host of different research questions. However, mining such publicly available data in a tailored fashion is currently not easy, as the available software tools typically require familiarity with the command-line interface, which constitutes a significant obstacle to many researchers. Additionally, different research groups utilize different software packages to perform LRS analysis, which often prevents a direct comparison of published results across different studies. To address these challenges, we have developed the Long-Read Analysis Pipeline for Transcriptomics (L-RAPiT), a user-friendly, free pipeline requiring no dedicated computational resources or bioinformatics expertise. L-RAPiT can be implemented directly through Google Colaboratory, a system based on the open-source Jupyter notebook environment, and allows for the direct analysis of transcriptomic reads from Oxford Nanopore and PacBio LRS machines. This new pipeline enables the rapid, convenient, and standardized analysis of publicly available or newly generated LRS datasets.

## 1. Introduction

Long-read sequencing (LRS), also known as third-generation sequencing, is a novel technology by which nucleic acids are sequenced as very large fragments or in the absence of any fragmentation. This provides a major advantage over conventional, short-read-based sequencing techniques, which rely on heavy fragmentation of DNA or RNA to a few hundred nucleotides and are typically limited to read lengths of approximately 150 nucleotides [1]. Among the various LRS platforms currently available, Oxford Nanopore Technologies and Pacific Biosciences of California (PacBio) have been adopted particularly widely [2]. LRS technologies can be utilized for a diverse range of applications, including the construction of novel genome assemblies, the identification and tracking of emerging virus variants, and the characterization of nucleotide methylation patterns [3,4]. In transcriptomics, the ability of LRS to sequence complete or nearly complete RNA transcripts has significantly improved the discovery, validation, and characterization of splice variants [5]. A recent analysis of human RNA LRS data deposited in the Genotype-Tissue Expression (GTEx) database identified over 70,000 novel transcripts and characterized shifts in splicing patterns based on allele-specific expression [6]. 

**Figure 1:**
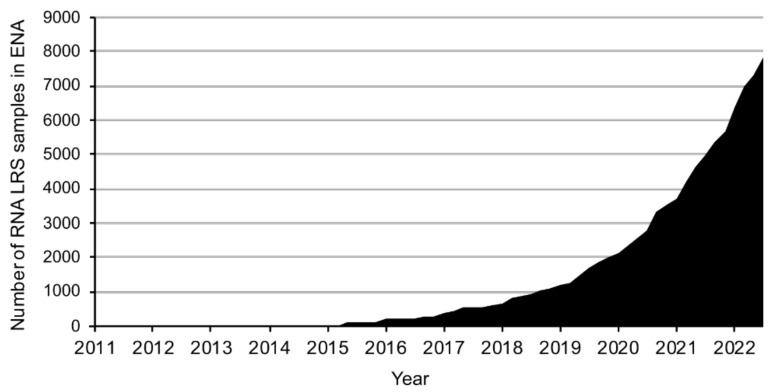
**The number of publicly available long-read RNA sequencing datasets has been increasing at an exponential rate.** Total number of long-read RNA sequencing samples deposited in the European Nucleotide Archive (ENA) over time. Sample selection criteria were (i) RNA sequencing, (ii) sequenced on an Oxford Nanopore or a PacBio machine, and (iii) containing more than 100,000 reads.

Given the unique advantages over other techniques, it is not surprising that the number of published LRS datasets has increased at an exponential rate (Figure 1). While publication of these data is usually accompanied by an analysis, the considerable number of different computational tools available to process LRS data has resulted in largely non-standardized analyses [7,8,9,10,11]. This often precludes direct comparison of data across different studies. Furthermore, answering new scientific questions with previously published LRS data may require a specific annotation set or a more recent reference genome not included in the original analysis. Thus, the ability to analyze LRS data from source files would likely benefit researchers from a wide variety of fields and backgrounds. However, the software packages used in the quality control, alignment, sorting, and read counting of LRS data typically require at least some use of a command-line interface, with which many researchers may not be familiar or comfortable, and depend on considerable computational power, exceeding what is typically available on standard computers found in many laboratories. There is therefore an unmet need for an easily accessible, free, user-friendly LRS analysis pipeline that can be used directly and without prior knowledge of programming languages. 

To address this need for researchers interested specifically in the analysis of long-read RNA sequencing data, we have developed the Long-Read Analysis Pipeline for Transcriptomics (L-RAPiT), the first cloud-based pipeline explicitly designed to implement existing LRS-specific RNA analysis tools in an easy-to-use format. L-RAPiT is built entirely on free and open-source software packages sourced from BioConda, GitHub, and long-read-tools.org [12,13,14]. It is designed to be fully compatible with the base version of the Google Colaboratory environment, which provides a web interface and cloud storage at no charge.

## 2. Results

### 2.1. Conceptual Framework

L-RAPiT aims to allow nearly anyone with an internet connection to perform a comprehensive analysis of long-read RNA sequencing data. To achieve this, the two primary goals were (i) to circumvent the need for high-end computational resources required by many LRS analysis programs and (ii) to create a user interface that requires little or no knowledge of the command-line interface, thus permitting researchers without bioinformatics expertise to perform the entire analysis process independently. We therefore designed L-RAPiT to be compatible with and entirely executable within the cloud-based, user-friendly Google Colaboratory environment, which typically provides at least 12 GB of random-access memory (RAM). As individual Google Colaboratory sessions are time-limited to 12 h, a minimum of approximately 10 million reads can be processed by the pipeline at a time, which in practice permits analysis of more than 99% of the LRS samples currently available in the public European Nucleotide Archive (ENA) repository. L-RAPiT was therefore designed to analyze a single sample per runtime and allow users to export output of interest to either a Google Drive account or a local hard drive. The pipeline includes all programs necessary to comprehensively characterize individual samples beginning with raw read data. We do not provide any infrastructure for performing differential expression analysis, differential transcript analysis, or differential splicing analysis, as these can generally be performed with the output from L-RAPiT on a standard lab computer utilizing well-documented software packages. 

### 2.2. User Experience

**Figure 2:**
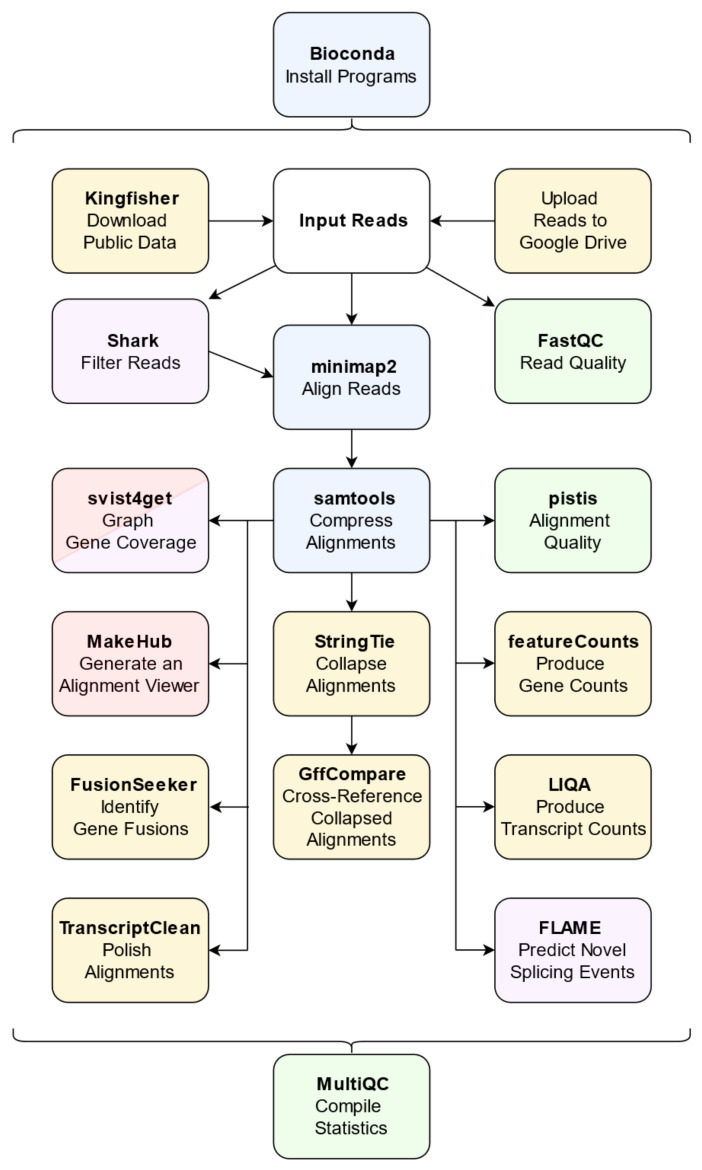
**Overview of L-RAPiT components and workflow.** Colors reflect the general purpose of each program: core elements of the pipeline are shown in blue; quality control programs in green; visualization programs in red; region-specific programs in purple; and other optional pipeline components in yellow. Arrows indicate the use of output from one program as input for another.

L-RAPiT has been optimized for use in conjunction with the Google Colaboratory environment, for which users only require a free Google Account. This approach renders the pipeline and all programs it contains entirely independent of the user’s operating system, thus precluding any compatibility issues during installation and use. After the user provides basic information about the desired analysis, such as the location of the raw data, the pertinent reference genome, and any genomic region of particular interest, the entire pipeline can be run by simply clicking through each step of the user interface. For each tool in the pipeline, the user can choose to export the output either to the associated Google Drive or a local hard drive. The computational processing itself is contained entirely within the Colaboratory environment, which cannot access the user’s Google Drive without the user’s explicit permission. Installation and use instructions are available on the GitHub page: https://github.com/Theo-Nelson/long-read-sequencing-pipeline (accessed on 12 December 2022). Use of any and all Google products or services must follow the Terms of Service put forth by Google. For more advanced users with sufficient computational resources, the entire pipeline can also be run locally as a stand-alone Jupyter notebook, independently of any Google products or services. L-RAPiT incorporates 18 established software packages, each of which performs a specific function (Table 1). This includes a core pipeline of three programs required for basic LRS data analysis, which can be supplemented with optional programs at the user’s discretion (Figure 2).

### 2.3. The Core Pipeline

L-RAPiT utilizes **BioConda** to install software packages and manage program-specific dependencies [14]. Alignment of raw sequencing data to the reference genome is performed with **minimap2**, which outputs a Sequence Alignment Map (SAM) file containing detailed information about each read, where it aligned in the reference genome, and how well it matches the reference sequence [18]. While SAM files are very informative and human-readable, they are also fairly large and unwieldy; therefore, many applications use Binary Alignment Map (BAM) files as their input. BAM files contain the same information as SAM files, but in a compressed format. The conversion from SAM files to BAM files is performed by **SAMtools**, the third core component, which also coordinate-sorts and indexes the files [19]. The resultant BAM files form the central product of L-RAPiT, as they can be used directly for a large number of downstream applications. This includes most of the optional L-RAPiT components described below and many other applications outside the scope of this pipeline, such as the Integrative Genomics Viewer (IGV) [31].

### 2.4. Optional Pipeline Components

#### 2.4.1. Source Data

The user can provide raw LRS data in two different ways: FASTQ files can be either uploaded manually, e.g., for unpublished data; or they can be downloaded directly from two public repositories, the ENA and the Sequence Read Archive (SRA) [32,33]. For the latter option, L-RAPiT provides the **Kingfisher** program, which automatically retrieves sequencing data based on the sample (“run”) identifier assigned by the corresponding repository [15].

#### 2.4.2. Transcript Quantification and Characterization

After alignment with minimap2, the read integration program **featureCounts** can be used to quantify expression at the gene level. The output is a simple text file listing the number of read counts for each gene in the annotation file, as well as a summary text file providing an overview of the success of the alignment and assignment of reads [22]. Additionally, L-RAPiT includes **LIQA**, a long-read-specific program for quantifying gene expression at the transcript level. The output is also a text file, containing the estimated number of assigned reads and the percentage of reads aligned to the particular transcript out of all reads aligning to the same locus [23]. Potential gene fusion events can be detected with **FusionSeeker**. The text file generated by this program lists the genes involved in the fusion event, along with the starting and ending coordinates of the segments [24]. LRS has a significantly higher error rate than conventional RNA-seq methods [34]. To mitigate this limitation, L-RAPiT offers the option of using **TranscriptClean** to correct mismatches, small insertions or deletions, and non-canonical splice junctions in the sequencing data [20]. The program produces new SAM and FASTA files with corrected read sequences, which can then be used for downstream applications that are sensitive to mismatches. Both the original alignments and those corrected by TranscriptClean can be provided as input to **StringTie** for *de novo* assembly into transcriptome annotations. The output file contains inferred information about gene structure and is organized in the standard Gene Transfer Format (GTF) [25]. This novel assembly can then be compared directly to the original reference assembly by utilizing **GffCompare**. This program generates detailed assembly statistics, annotated GTF files, and transcript novelty classifications [26].

#### 2.4.3. Region-Specific Analysis 

L-RAPiT allows users to focus on a particular gene locus instead of the entire annotated genome. This permits the rapid analysis of a large number of datasets, which can be used, for instance, to quickly identify tissues that express a specific transcript variant of a gene of interest. Prior to alignment, the **Shark** program identifies and isolates only those reads that are relevant to the gene locus in question and compiles them into a new FASTQ file. This can then be used as input for minimap2 and run through the remaining L-RAPiT pipeline [17]. For users that require alignment of their sample to the full genome but are interested in detecting novel splicing patterns at only a small number of gene loci, **FLAME** can be used to selectively probe a particular genomic location after alignment. FLAME separates reads into those that match the provided annotation file and those that are incongruent with it; quantifies the incongruent reads; identifies potential splice sites; and generates a Browser Extensible Data (BED) file that can be used to directly visualize the incongruent reads with IGV or other genome visualization tools [21].

#### 2.4.4. Region-Specific Visualization

As mentioned above, the indexed BAM files generated by minimap2 and SAMtools can also be imported directly into general genome viewer programs such as IGV to visualize aligned reads [31]. For users primarily interested in one particular genomic locus, e.g., the expression levels or splicing behavior of a specific gene, L-RAPiT provides the option of generating an immediate snapshot of that locus with **svist4get**. This program generates a PDF and a PNG file with a graphic representation of the genomic region along with its chromosomal coordinates, annotated transcript variants, and any aligned reads present in the analyzed dataset [27]. 

#### 2.4.5. Transcriptome-Wide Visualization

For advanced visualization of the analyzed LRS data in a broader context, L-RAPiT also incorporates MakeHub, which permits full integration into the UCSC Genome Browser [29,35,36]. MakeHub generates all the files necessary to upload the analyzed LRS data as a custom track to the UCSC Genome Browser from a publicly accessible web server.

#### 2.4.6. Quality Control

L-RAPiT includes three separate quality-control (QC) programs, each designed for a different purpose. The **FastQC** program generates basic quality-control metrics about the raw LRS data, such as information about the sequence quality, GC content, etc. [16]. **Pistis** has a significantly longer processing time than FastQC, but it provides a more sophisticated analysis that includes the relationship between base quality and read length, as well as the distribution of the percentage of bases per read that were identical to the reference index [28]. **MultiQC**, the third and final QC component of L-RAPiT, summarizes the output of a large number of programs within a single HTML file and is therefore most useful when run last [30]. If the entire L-RAPiT pipeline has been run, the MultiQC output includes general statistics of the alignment, such as the percentage of aligned reads, GC content, and percentage of duplications; a bar graph summarizing the featureCounts output; graphs summarizing transcript assembly statistics; and visual summaries of 11 different FastQC metrics, including an overall status check to indicate whether the results are within generally acceptable parameters.

### 2.5. Use Case: Discovery and Validation of Novel Splice Variants 

L-RAPiT incorporates several tools that are designed to characterize splicing events at a specific gene locus. Below, we provide an example of such an analysis and consider different usage scenarios. The use case focuses on the core pipeline before discussing the optional components.

#### 2.5.1. Background

This use case focuses on long non-coding RNAs (lncRNAs), defined as RNA transcripts that are at least 200 nt in length and are not translated into proteins [37]. Current estimates suggest that at least 19,933 lncRNA genes are present in the human genome and give rise to 57,936 different transcripts (GENCODE release v42) [38]. Only a small part of these non-protein-coding genes has been thoroughly investigated so far; nonetheless, various lncRNAs have already been implicated as important regulators of a wide variety of essential biological processes, including cellular proliferation, development, nuclear organization, and immunity [39,40,41,42]. Among these is the lncRNA *LINC00173*, which has been reported to be involved in granulocyte development and cytokine expression, as well as a broad range of malignancies and other pathologies [43,44,45,46,47,48,49,50,51,52,53,54,55,56,57,58,59,60]. While compelling evidence for the importance of the *LINC00173* gene locus in some of these observations has been presented, a consensus on the underlying molecular mechanism has still not emerged, in large part because it remains unclear which individual transcripts arise from this locus [43,44]. In this use case, we therefore endeavored to leverage LRS data to better characterize the transcript variants that are encoded by the *LINC00173* gene.

#### 2.5.2. Sample History

Our previous work has shown modest but robust expression from the *LINC00173* locus in the human HEK 293T cell line [44]. We therefore searched for a publicly available RNA LRS dataset derived from this cell line and identified a recent study by Brannan et al. which aimed to map RNA interaction partners of RNA-binding proteins and ribosomes [61]. Importantly, the study included a control sample with a relatively large number of sequencing reads from HEK-293T-derived cDNA, which we selected for analysis with L-RAPiT (accession number: SRR12389274).

#### 2.5.3. Input, Installation, and Data Retrieval

After opening L-RAPiT by clicking on the link provided on the GitHub page, the following settings were entered manually in the section “Parameter Input and User Instructions” (user input is formatted in bold and italics for emphasis):

First cell:

Designating the name of the output directory (default setting):


  %env PIPELINE_FILE_PATH=***/content***


Fourth cell:

Providing the accession number for the sample of interest (see above):


  %env ACC=***SRR12389274***			


Providing the reference genome (default setting, human genome version GRCh38/hg38):


  %env INDEX_FILE_PATH=${PIPELINE_FILE_PATH}/long-read-sequencing-pipeline/prebuilt_indices/***hg38.fa***


Providing the genome annotation file (default setting, Ensembl file for GRCh38/hg38):


  %env ANNOTATION_FILE_PATH=${PIPELINE_FILE_PATH}/long-read-sequencing-pipeline/prebuilt_indices/***hg38.ensGene.gtf***


Setting the genome coordinates for *LINC00173* in GRCh38/hg38 (located on chromosome 12, bases 116,533,422-116,536,513). Note that Ensembl annotation requires the addition of “chr” in front of the chromosome number, whereas NCBI annotation only uses the number itself (i.e., “chr12” and “12”, respectively):


  %env CHROMOSOME=***chr12***
  %env CHROMOSOME_START=***116533422***
  %env CHROMOSOME_FINISH=***116536513***


Providing a single-word identifier for the region of interest, such as the name of the gene locus. Note that for FLAME to run as intended, this name must match the gene name in the genome annotation file:


  %env REGION_NAME=***LINC00173***


Providing name and contact information for MakeHub tracks, if desired:


  %env HUB_KEYWORD=***LINC00173***
  %env HUB_NAME=***“Human LINC00173”***
  %env HUB_EMAIL=***your@email.address***


After entering these parameters, they were submitted to the pipeline by clicking the four individual cells (or by collapsing the section and clicking on the summary cell). Next, the Google Drive of an associated account, into which the system was logged at the time, was mounted by clicking the summary cell of the section, “Mounting your Google Drive/Exporting to Your Local Hard Drive”. All pipeline programs were then installed to the Google Colaboratory by clicking the summary cell of section “BioConda: Package Installations”. Next, the FASTQ file containing the raw LRS data of our sample of interest, SRR12389274, was automatically downloaded after clicking the summary cell in the section “Kingfisher: procurement of sequence files—usage”. Finally, the reference genome was downloaded and installed by clicking the summary cell in the section “Reference Genome—installation”. 

#### 2.5.4. Read Filtering

As this use case focuses on one particular gene, *LINC00173*, we employed Shark to isolate those reads within the dataset that likely derive from this locus. We used the L-RAPiT default parameters for both confidence level (option -c .40) and k-mer size (-k 10). Increasing either c or k will increase the stringency of the filter, whereas decreasing either value will allow more reads to pass through it. Users can directly adjust these values in the third cell of this section if they are not satisfied by the default filter results [17]. The filtered FASTQ file produced by Shark is automatically designated as the combination of the user-provided region name and the sample number, i.e., LINC00173SRR12389274 in this example. Importantly, the Shark usage section of L-RAPiT automatically alters the ACC variable, which provides the pipeline with the location of the source data, to this designation. Therefore, if Shark is utilized, L-RAPiT will by default continue with the filtered reads instead of the full dataset. This process drastically reduces the number of reads to be analyzed, often substantially decreasing the time required to run the remainder of the pipeline. As *LINC00173* is expressed at comparatively low levels, only approximately 0.1% of raw reads passed the filtering criteria in the use case.

#### 2.5.5. Alignment

Alignment is the most computationally complex step of sequencing analysis pipelines [62]. L-RAPiT employs minimap2 for this task. Two separate steps are necessary to run this program within the Google Colaboratory environment: index minimization and the alignment itself. Minimap2 offers multiple presets for different types of transcriptomic data. Below, we discuss the three most relevant presets based on their assumptions and how to alter the default setting, if desired by the user. The decision to apply a non-default option should be based on the sequencing protocol under which the sample of interest was generated. 

##### L-RAPiT Default Setting: Spliced Reads

L-RAPiT assumes that the user has selected a sample of transcriptomic origin, which is characterized by the presence of RNA splicing patterns. In the minimap2 usage module, the command option -x splice instructs the aligner to consider canonical splicing signals [18]. This is the L-RAPiT default setting and should not be altered when analyzing publicly available samples with incomplete information on the underlying sequencing protocol. 

##### Setting for High-Quality Sequences

For samples generated with a protocol permitting higher base calling accuracy, such as the PacBio CCS workflow, a preset with stricter scoring metrics can be applied. In such a case, the minimap2 usage module command can be altered to the option -x splice:hq. This increases the sensitivity to small exons and improves the overall accuracy, but it also increases the risk of introducing false small introns. 

##### Setting for Nanopore Direct RNA-seq

For samples produced by direct RNA sequencing, i.e., without a cDNA intermediate, a lower k-mer size can be utilized. In this case, the commands in both the minimap2 index minimization module and the minimap2 usage module need to be altered to option -k14 from the default value -k15. The -x splice option in the usage module should remain unchanged. These settings improve the sensitivity of alignments to the first and last exon of transcripts.

##### Use Case

The use-case sample, SRR12389274, was generated by direct cDNA nanopore sequencing, eliminating the need to alter the k-mer size. There is also no indication that a high-quality protocol was used to minimize or correct base calling errors [61]. Therefore, we used the L-RAPiT default settings to align the Shark-filtered reads to the human genome by clicking directly on the summary cells of the minimap2 index minimization and usage sections. The SAM file produced by minimap2 was then converted to an indexed BAM file by clicking the summary cell of the section “samtools: Write/Index SAM to BAM—usage”. To export the BAM file and the corresponding BAI index file, we clicked the summary cell of the section “samtools: Write/Index SAM to BAM—export”.

##### Evaluation and Experimental Validation

**Figure 3:**
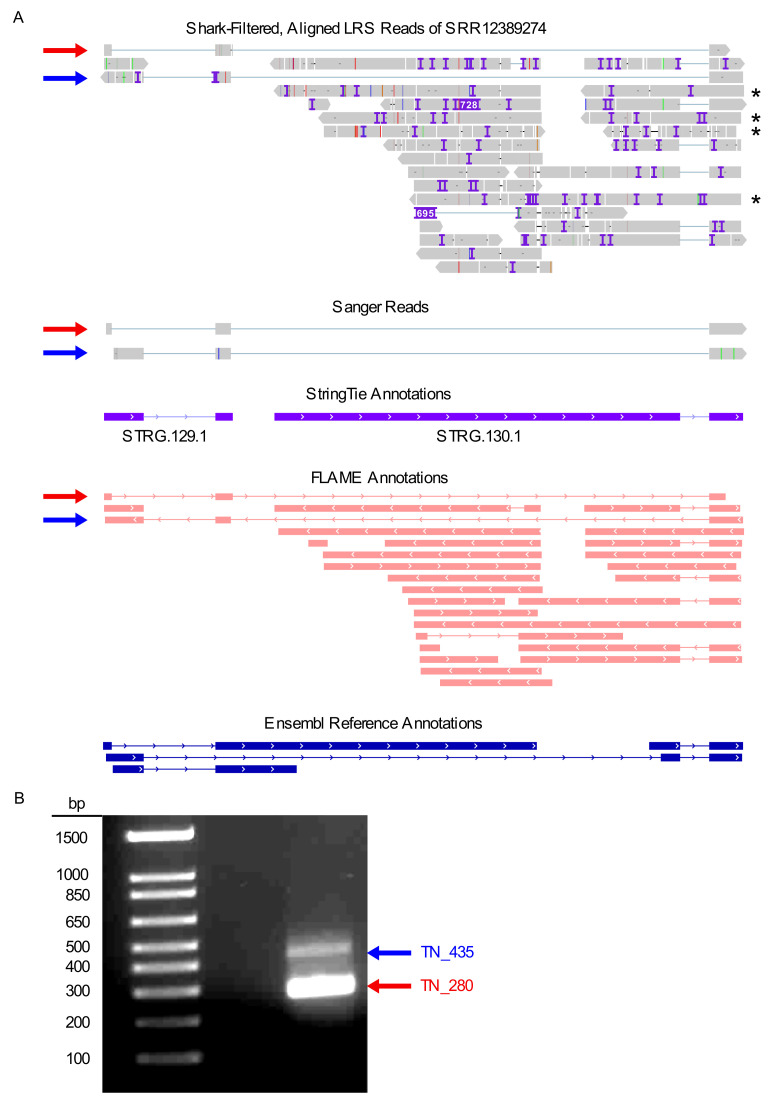
**L-RAPiT identifies novel transcript variants at the *LINC00173* locus in the use-case sample.** (**A**) Visualization of L-RAPiT output for the use case at the *LINC00173* locus. From top to bottom: reads filtered by Shark, aligned by minimap2, and compressed and indexed by SAMtools (asterisks indicate reads with retained introns); experimental validation of TN_280 and TN_485 sequences, obtained by PCR and subsequent Sanger sequencing (note that TN_280 was sequenced from both ends, whereas TN_485 was only sequenced from the 5′ end, resulting in a truncated 5′ sequence); StringTie-generated *de novo* annotation of the LRS alignment; FLAME-generated comparison of the LRS alignment with the reference annotation (salmon color indicates no matching transcript in the reference); and the reference transcripts currently annotated in Ensembl. Image generated with IGV. (**B**) Gel image of PCR-amplified transcripts TN_280 and TN_435. Ladder in the first lane indicates size in bp. Throughout the figure, red arrows indicate TN_280; blue arrows indicate TN_485.

To visualize the use-case alignment in the context of current genome annotations, we viewed the exported BAM and BAI files with IGV, a stand-alone program that can be downloaded for Mac, Windows, and Linux platforms at no cost. As our analysis was confined to the *LINC00173* locus, we selected its genomic coordinates (chr12: 116,533,422-116,536,513 in GRCh38/hg38; note that coordinates shift between different genome versions). Interestingly, the read alignments output by minimap2 did not recapitulate the annotated transcript variants precisely (Figure 3A). In general, such differences may represent either technical artifacts or true biological differences. All RNA-seq methods, in particular nanopore technologies, are susceptible to artifacts resulting from 5′ truncation [63,64]. Therefore, reads that overlap with the reference transcript in the 3′ portion but are shorter at the 5′ end may reflect the same transcript rather than separate transcript variants with different transcriptional start sites (TSS). Conversely, reads with a 5′ end extending beyond the annotated transcript may indicate that the corresponding TSS in the reference transcriptome is incorrect. The use-case alignment features several examples of both of these phenomena. Additionally, four reads appeared to retain an intron near the 3′ end of the *LINC00173* locus. It is unclear whether these represented novel, mature lncRNA transcripts or pre-lncRNA transcripts that were yet to be fully spliced. Intriguingly, two reads contained a unique exon, consisting of approximately the first 80 bases of the annotated second exon of the RefSeq transcript NR_027345.1. This suggested the existence of two previously unreported *LINC00173* splice variants. Based on their respective length, we designated these potential novel transcripts as TN_280 and TN_435 (Figure 3A). To determine whether these reads reflected a technical artifact or did indeed represent novel splice variants of the *LINC00173* locus, we designed primers matching the start and end points of the transcripts as predicted by the minimap2 alignment. Conventional PCR on cDNA from HEK 293T cells yielded two bands of the expected sizes (Figure 3B and Supplementary Figure S1). Sanger sequencing confirmed that these PCR products were indeed matches for the novel transcript variants TN_280 and TN_435 predicted by L-RAPiT (Figure 3A). Further experiments will be needed to establish the precise TSS for these splice variants; however, the comparatively short length and the identical 5′ ends of TN_280 and TN_435 make 5′ truncation in the LRS sample a fairly unlikely possibility.

#### 2.5.6. Annotation

Next, we used StringTie to collapse the aligned reads into a miniature reference transcriptome for the *LINC00173* locus by clicking the summary cell in the module “StringTie: transcript assembly—usage”. We then exported the resulting GTF annotation file via section “StringTie: transcript assembly—export” and visualized the *LINC00173* locus in IGV (Figure 3A). The default parameters of StringTie require at least 1.5 reads of a given transcript to be detected for inclusion in the annotation. As TN_280 and TN_435, although experimentally validated, were each represented by only a single read in the use-case sample, they were not included in the *de novo* annotation. Instead, StringTie generated a consensus sequence for two transcripts, designated as STRG.129.1 and STRG.130.1, neither of which matched the established RefSeq or Ensembl transcript annotations for *LINC00173*. While these results may reflect a distinct splicing profile at this locus in HEK 293 T cells, it more likely illustrates the limitations of StringTie in the context of genes with robust but comparatively low expression levels. To further corroborate this hypothesis, we performed a separate, analogous analysis of the highly expressed gene *GAPDH* in the same use-case sample (SRR12389274; chr12: 6,534,517-6,538,371). With these parameters, StringTie generated a *de novo* annotation that closely matched the Ensembl reference annotation (Supplementary Figure S2).

To perform an objective comparison between the *LINC00173* annotation generated by StringTie and the reference, we used the GffCompare module in L-RAPiT. Table 2 shows the output of the .tracking file generated by GffCompare, which provides a direct comparison of transcripts in a *de novo* annotation and a reference annotation. Of note, GffCompare determines identity between transcripts only on the basis of the intron structure; the length of the first and the final exon are not considered, an approach that aims to minimize noise from 5′ and 3′ truncation events. As the only intron present in STRG.129.1 and STRG.130.1 is shared with at least one reference transcript, GffCompare assessed them to be equivalent to the reference annotation, notwithstanding the large differences in size of the first and last exon in both cases (Figure 3A and Table 2). Conversely, the output generated by executing the FLAME module, which analyzes the reads at a specific locus rather than across the whole genome, classified all reads at the *LINC00173* locus as incongruent with the reference annotation (Figure 3A). These contradictory results produced by different tools emphasize the importance of the visual inspection of a locus of interest when the discovery of novel transcript variants is a main objective, especially in cases with low expression or samples with few reads.

#### 2.5.7. Read Correction

**Figure 4:**
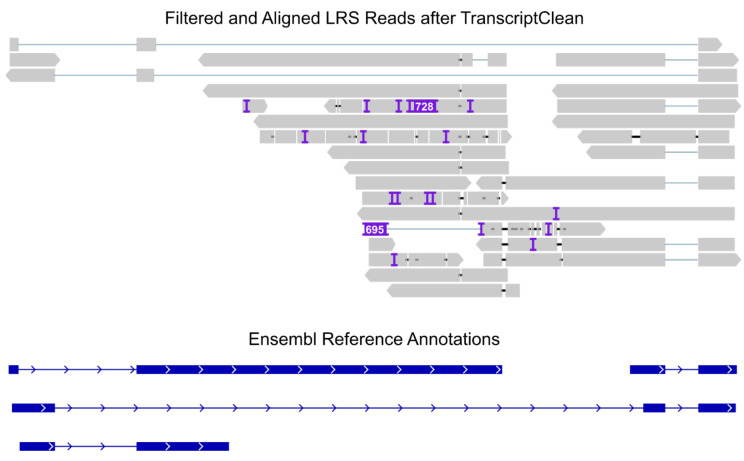
**TranscriptClean generates polished reads that permit a cleaner alignment.** Alignment of Shark-filtered reads to the *LINC00173* locus after processing by TranscriptClean and compression by SAMtools. Processed reads are shown in gray; transcripts currently annotated in Ensembl are shown in blue. Image generated with IGV.

To obtain a polished set of aligned reads, we activated the TranscriptClean usage module, which directly pipes read-corrected SAM files into SAMtools to generate indexed BAM files. These were downloaded with the corresponding TranscriptClean export section and visualized with IGV (Figure 4). Compared to the original BAM files, TranscriptClean-polished reads provided a far cleaner alignment. However, the correction process carries a high risk of introducing biases and artifacts that could confound subsequent analyses of the alignment; therefore, L-RAPiT by default only utilizes the original BAM files outside the TranscriptClean module itself.

#### 2.5.8. Read Counting

To count the reads aligned to the *LINC00173* gene locus, we activated the featureCounts usage module. The generated .summary file indicated that a total of 1883 reads had been assigned a gene in the analysis. The count matrix for all genes in the reference genome, contained in the corresponding .txt file, placed 28 of those reads at the *LINC00173* gene (Ensembl gene identifier ENSG00000196668). The large number of reads that mapped to genomic locations other than *LINC00173* reflects the comparatively low stringency of the L-RAPiT default setting for read filtering by Shark, which maximizes the potential for discovery of novel transcript variants. Activating the LIQA usage section, we next sought to quantify individual transcripts. This approach reported 21 total reads distributed over two of the *LINC00173* transcript variants annotated by Ensembl (Table 3). The high similarity between the total read count of featureCounts and LIQA underscores the robustness of this approach. We did not detect any gene fusion events in our dataset with FusionSeeker, consistent with the complete alignment of all included reads to the *LINC00173* locus (Figure 3A).

#### 2.5.9. Visualization

**Figure 5:**
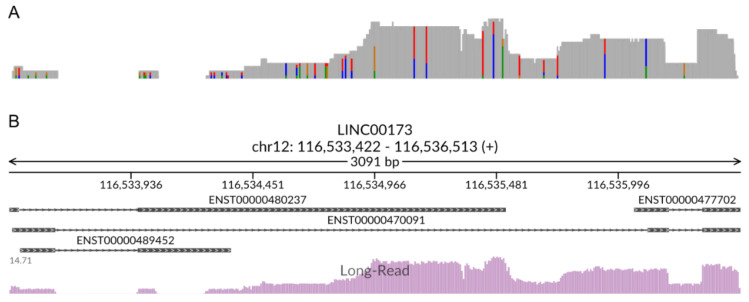
**svist4get rapidly generates a coverage map for a locus of interest.** (**A**) The *LINC00173* coverage map visualized with IGV compared to (**B**) the locus-specific coverage map produced by svist4get.

Executing the svist4get usage module generated a coverage chart for the *LINC00173* locus that generally mirrored the results of the IGV visualization (Figure 5). This tool is primarily beneficial to users who employ L-RAPiT without Shark-mediated read filtering, as it is a convenient way to quickly visualize a region of interest while still analyzing all reads in the input dataset. Running MakeHub produced the files necessary to set up a custom UCSC track hub, which could be hosted on a publicly accessible server [36].

#### 2.5.10. Quality Control

**Figure 6:**
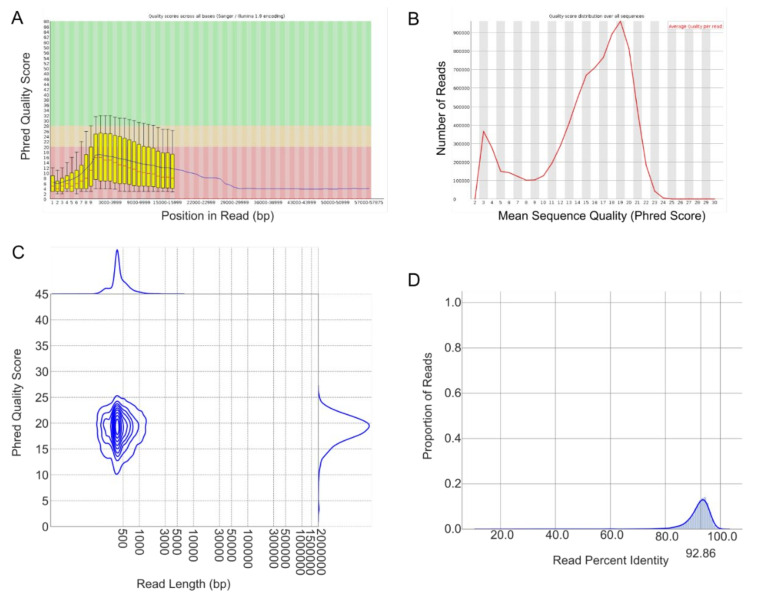
**L-RAPiT provides detailed quality-control metrics before and after alignment.** (**A**,**B**) Selected output from FastQC for the unprocessed, unaligned reads of use-case sample SRR12389274. (**A**) Box plot showing the average Phred score and its spread for ranges of nucleotide position within reads. (**B**) Graph of the average Phred score per read and corresponding frequency. (**C**,**D**) Selected output from Pistis for the use-case sample after Shark filtering and minimap2 alignment. (**C**) Density plot of read length vs. the average Phred score per read, as well as the associated distributions. (**D**) Read alignment percent identity and the proportion of reads with that percentage.

In order to assess the data quality, we executed the three quality-control modules included in L-RAPiT. FastQC generated an HTML file that could be opened with a standard internet browser and provided basic metrics on the unaligned reads in the FASTQ file. Phred scores (*Q*) provide an estimate of the probability *P* that a given base call is wrong, where *Q* = −10·log_10_(*P*). FastQC was originally designed to analyze short-read sequencing data, which generally have far higher quality scores than LRS datasets. While short-read data typically have Phred scores well above 35, LRS data tend to fall between 10 and 20 or even lower. Therefore, FastQC considers even LRS reads of comparatively high quality to be poor. Nonetheless, FastQC provides valuable information about potential technical problems, in particular large variations in quality between samples or a heavily skewed mean GC content per read. FastQC analysis of the use-case sample showed robust average Phred scores above 10 even for very long reads exceeding 20,000 nucleotides, but also a significant number of poor-quality reads with scores between 3 and 5 (Figure 6A,B).

Unlike FastQC, Pistis is specifically designed to perform quality assessment of LRS data, before and after alignment. Of note, if L-RAPiT is run in the default order of tools and the user chooses to utilize Shark, FastQC will provide quality assessment of the original input sample, whereas Pistis will analyze the Shark-filtered reads. By default, Pistis randomly samples 50,000 reads from the input sample and aborts if fewer reads are provided. As the summary output of featureCounts indicated that fewer than 10,000 reads had remained in our sample after Shark filtering, we restricted sampling to 4000 reads by manually adding the option --downsample 4000 to the Pistis usage module. The output of Pistis contained a density plot that visualized the distribution of read length and Phred score across the full spectrum of reads, which indicated that the reads filtered from the original input data were predominantly of sound quality with Phred scores between 10 and 25 (Figure 6C). Pistis furthermore produced a graph of the read alignment percent identity, which plots the percentage of nucleotides in each read that are identical to the reference sequence and the proportion of reads with that percentage. This metric also did not indicate any technical issues with the sample or the alignment and indeed recorded a fairly high average alignment identity peaking at more than 90% (Figure 6D).

Finally, we activated the MultiQC section, which compiled the reports produced by FastQC and other pipeline programs including featureCounts and GffCompare, thus generating a convenient overview document [31]. 

## 3. Discussion

Next-generation sequencing (NGS) has rapidly become a central component of modern molecular biology. One particularly advantageous aspect of NGS datasets is that they can be queried by the scientific community to address questions unrelated to the study with which they were originally published, such as understanding splicing patterns in different tissues or establishing regulatory networks for any gene of interest. The full realization of this remarkable potential, however, has been hampered by two significant obstacles: (i) most classically trained biologists have only a limited understanding of computational sciences and therefore lack the skills necessary to operate the various software packages required to analyze sequencing data quickly and independently; and (ii) many of these packages depend on high-end computational hardware that is not available to all scientists. Several solutions have been developed to address these problems for conventional, short-read-based NGS datasets, such as the cloud-based Galaxy suite [65]. While Galaxy recently incorporated a set of tools designed for Oxford Nanopore LRS analysis, a generalized, transcriptomics-focused LRS analysis pipeline has, to our knowledge, not been published previously [66].

L-RAPiT contains a selection of open-source tools compatible with both Oxford Nanopore and PacBio systems that permit a comprehensive analysis of LRS transcriptomic datasets. While the underlying Jupyter notebook structure technically still employs written commands, the vast majority of parameters is preset for the user, allowing for a mostly click-based pipeline experience. Simultaneously, the immediate accessibility of the written commands provides interested users with an opportunity to gain initial exposure to command-line interfaces and may even serve as an educational tool. Integrating L-RAPiT into the Google Colaboratory platform, which is available free of charge, renders the pipeline completely independent of the user’s (i) computational resources and (ii) operating system, thus eliminating any risk of hardware bottlenecks, version conflicts, or installation complications. However, use of the Google Colaboratory, while certainly convenient, is not a technical requirement for L-RAPiT.

Within L-RAPiT, the user can choose between a global transcriptomic analysis of the entire LRS sample input or a more limited analysis that focuses exclusively on a user-selected genomic region. This latter feature significantly reduces the overall pipeline run time, which may be advantageous to users aiming to characterize a specific gene locus or chromosomal region in a large number of samples. Both of these approaches produce the same type of output, including compressed sequence alignment files (BAM), which can be used by a variety of different downstream applications within and outside of L-RAPiT, and gene- or transcript-level read counts generated by featureCounts and LIQA, respectively, which can be used directly with DESeq2, edgeR, or similar programs to determine differential gene expression.

The use case presented here illustrates some of the strengths and weaknesses associated with L-RAPiT: Our focused analysis of the *LINC00173* locus identified two previously unreported transcript variants, whose physical existence in independent samples we were able to confirm readily by PCR and Sanger sequencing. However, the conflicting results generated by StringTie, GffCompare, and FLAME demonstrate the shortcomings of automated annotation, at least in the context of individual loci with robust but low expression levels. Importantly, these findings also emphasize the tremendous potential of LRS data in establishing more accurate transcriptome annotations than are currently available even from tightly curated and reliable sources such as RefSeq and Ensembl.

## 4. Materials and Methods

### Experimental Validation of Novel Splice Variants in the Use Case

HEK 293T/17 cells (ATCC, Manassas, VA, USA) were maintained in DMEM supplemented with 10% fetal bovine serum (both Gibco/Thermo Fisher Scientific, Waltham, MA, USA) in a humidified incubator at 37 °C and 5% CO_2_. Resting cells were lysed directly in Buffer RLT with β-mercaptoethanol. Total RNA was isolated using the RNeasy Mini Kit (Qiagen, Hilden, Germany) according to the manufacturer’s instructions, including the recommended on-column DNase I digest. RNA was reverse-transcribed into cDNA using oligo(dT)_12–18_ primers and the SuperScript III Reverse Transcriptase (Thermo Fisher Scientific). PCR to detect the two novel transcript variants was performed using the GoTaq 2X MasterMix (Promega, Madison, WI, USA), also according to the manufacturer’s instructions, with primers of the sequence 5′-CGAGGCTCCCACCTGCTCTAAGC-3′ and 5′-TGCCAGAGTGACTGGGAGTTTATTTGG-3′. Results were visualized by standard electrophoresis with ethidium bromide-containing agarose gels. Visible bands were excised, DNA was purified with the QIAquick Gel Extraction Kit (Qiagen), and Sanger sequencing was performed by Genewiz/Azenta Life Sciences (Chelmsford, MA, USA).

## Figures and Tables

**Table 1:** Individual programs incorporated into L-RAPiT and their function.

Program	Function	Importance	Ref.
Google Drive	permanent cloud storage	optional	n/a
**BioConda**	**bioinformatics software package manager**	**required**	[14]
Kingfisher	download sequencing files from the European Nucleotide Archive and Sequence Read Archive	optional	[15]
FastQC	initial sequencing data quality report	optional	[16]
Shark	region-specific read filtering	optional	[17]
**minimap2**	**read alignment**	**required**	[18]
**SAMtools**	**sort and compress alignments**	**required**	[19]
TranscriptClean	remove insertions/deletions from alignments	optional	[20]
FLAME	region-specific characterization of splicing patterns	optional	[21]
featureCounts	read quantification	optional	[22]
LIQA	transcript quantification	optional	[23]
FusionSeeker	detection of gene fusion events	optional	[24]
StringTie	transcript assembly	optional	[25]
GffCompare	transcript assembly statistics	optional	[26]
svist4get	region-specific coverage visualization	optional	[27]
Pistis	post-alignment quality control	optional	[28]
MakeHub	interactive read viewer	optional	[29]
MultiQC	combined quality-control output	optional	[30]

**Table 2:** Output of GffCompare for the use case, comparing the results of StringTie to the Ensembl reference annotation.

Transcript ID	Gene ID	Reference Gene| Reference Transcript	Code	Supporting Evidence
TCONS_00000106	XLOC_000101	ENSG00000196668|ENST00000489452	=	q1:STRG.129|STRG.129.1|2|913.430603|754.408752|1.681929|273
TCONS_00000107	XLOC_000101	ENSG00000196668|ENST00000477702	=	q1:STRG.130|STRG.130.1|2|2806.789307|2318.147217|5.168231|2128

**Table 3:** Transcript counts for the use case generated by LIQA.

Ensembl Gene ID	Ensembl Transcript ID	Transcript Count	% of Total Gene Count	Confidence
ENSG00000196668	ENST00000477702	9	0.42857143	0.66666667
ENSG00000196668	ENST00000489452	0	0	0.66666667
ENSG00000196668	ENST00000480237	12	0.57142857	0.66666667
ENSG00000196668	ENST00000470091	0	0	0.66666667

## Data Availability

All sequencing data utilized in this study are publicly available as described in the manuscript.

## Supplementary Materials

The following supporting information can be downloaded at: https://www.mdpi.com/article/10.3390/ijms232415851/s1.
