# Supplementary material for: L-RAPiT: A Cloud-Based Computing Pipeline for the Analysis of Long-Read RNA Sequencing Data
Source: Int J Mol Sci. 2022 Dec 13;23(24):15851. doi: 10.3390/ijms232415851 (PMC9781625; doi:10.3390/ijms232415851)
Supplement: Supplementary file 1 [file ijms-23-15851-s001.zip › FigureS1.pdf]

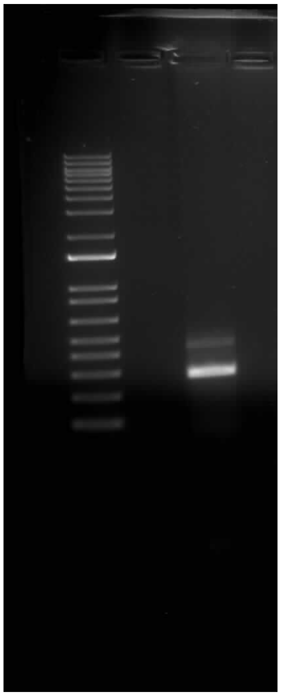

Supplementary Figure S1. L-RAPiT identifies novel transcript variants at the *LINC00173* locus in the use-case sample. Uncropped gel image of PCR-amplified transcripts TN_280 and TN_435. See Figure 3 for details.
