# Supplementary material for: L-RAPiT: A Cloud-Based Computing Pipeline for the Analysis of Long-Read RNA Sequencing Data
Source: Int J Mol Sci. 2022 Dec 13;23(24):15851. doi: 10.3390/ijms232415851 (PMC9781625; doi:10.3390/ijms232415851)
Supplement: Supplementary file 1 [file ijms-23-15851-s001.zip › FigureS2.pdf]

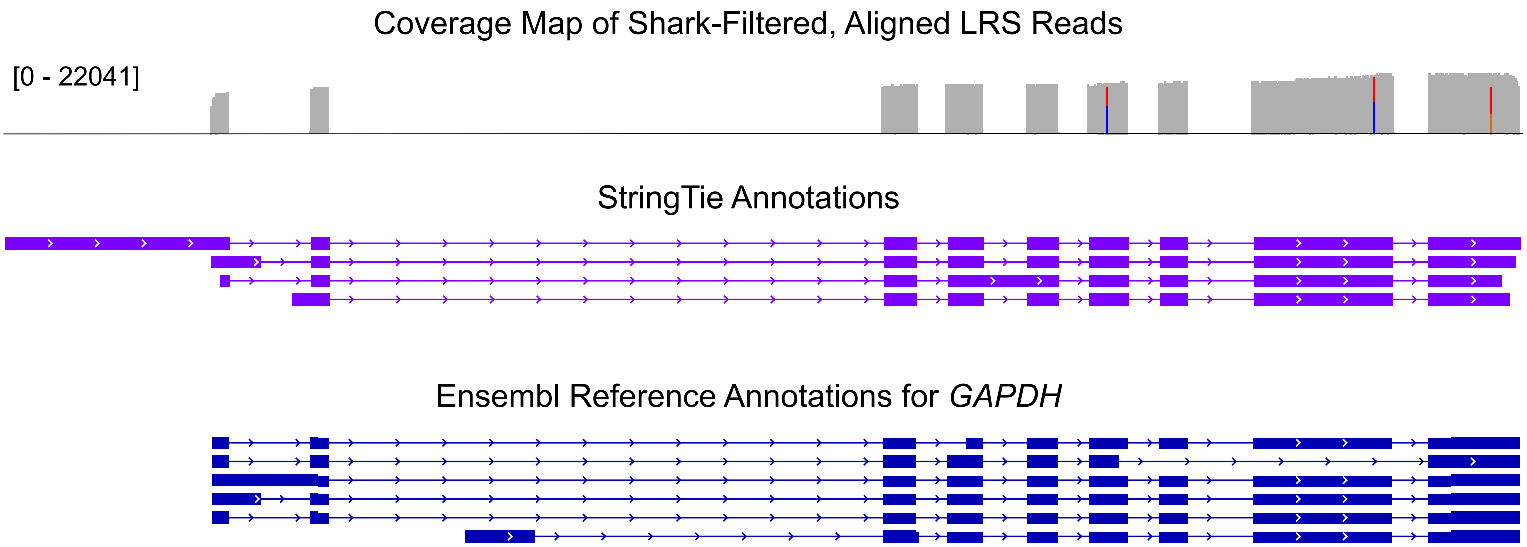

Supplementary Figure S2. StringTie results for *GAPDH*, a highly expressed gene. Separately from the use case analyzing *LINC00173*, L-RAPiT was run with the same parameters but targeting the gene *GAPDH* (chr12: 6,534,517-6,538,371), which is expressed at high levels in HEK 293T cells. From top to bottom: coverage map of the *GAPDH* locus showing reads filtered by Shark, aligned by minimap2 and compressed and indexed by SAMtools; StringTie-generated *de novo* annotation of the LRS alignment; and the reference transcripts currently annotated in Ensembl. Image generated with IGV.
